# Supplementary material for: Saturated very long chain fatty acid configures glycosphingolipid for lysosome homeostasis in long-lived C. elegans
Source: Nat Commun. 2021 Aug 20;12:5073. doi: 10.1038/s41467-021-25398-6 (PMC8379269; doi:10.1038/s41467-021-25398-6)
Supplement: Supplementary file 1 — Supplementary Information File [file 41467_2021_25398_MOESM1_ESM.pdf]

Supplementary information

Saturated very long chain fatty acid configures glycosphingolipid for lysosome homeostasis in long-lived *C. elegans*

Feng Wang[1,4], Yuxi Dai[1,4], Xufeng Zhu[2], Qilong Chen[3], Huanhu Zhu[2], Ben Zhou[3], Haiqing Tang[1,*] and Shanshan Pang[1,*]

[1]School of Life Sciences, Chongqing University, Chongqing, 401331, China

[2]School of Life Science and Technology, ShanghaiTech University, Shanghai, 201210, China

[3]CAS Key Laboratory of Nutrition, Metabolism and Food Safety, Shanghai Institute of Nutrition and Health, University of Chinese Academy of Sciences, Chinese Academy of Sciences, 200031, China

[4]These authors contributed equally

[*]Correspondence: hqtang@cqu.edu.cn (H.T.), sspang@cqu.edu.cn (S.P.)

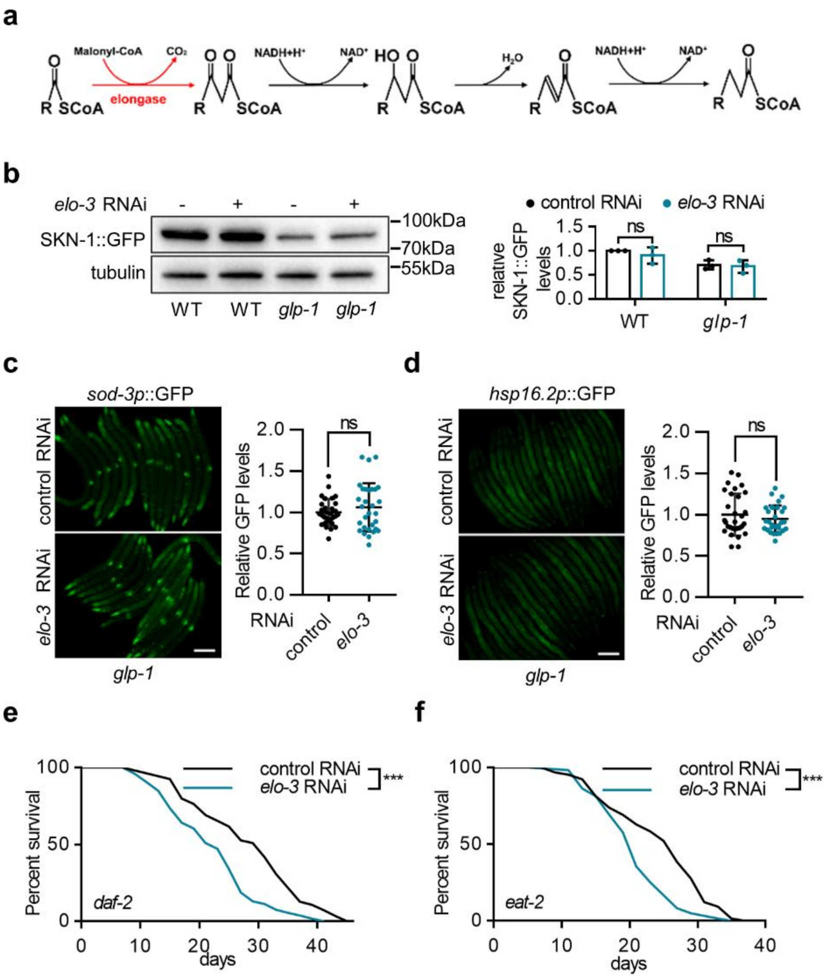

Supplementary Fig. 1. Effects of ELO-3 on the expression of DAF-16 and HSF-1 reporters, and the lifespan of other longevity models. (a) Schematic of fatty acid elongation pathway. **(b)** Effects of *elo-3* RNAi on the protein levels of SKN-1::GFP measured by immunoblot analysis. n = 3 independent experiments. **(c-d)** Effects of *elo-3* RNAi on the expression of *sod-3p*::GFP **(c)** and *hsp-16.2p*::GFP **(d)** in *glp-1* mutants. n = 30 animals. **(e-f)** Effects of *elo-3* RNAi on the lifespan of *daf-2* **(e)** and *eat-2* **(f)** mutants. Data are represented as mean ± SD. ****p* < 0.001. Fig. s1b was analyzed by two-way ANOVA with Turkey's multiple comparison test. Figs. s1c and s1d were analyzed by unpaired two-tailed t test. See Supplementary Table 2 for statistical analysis and additional repeats of the survival assays **(e, f)** that were analyzed by log-rank (Mantel-Cox) test. Scale bar = 100 µm for panels c and d. Source data are provided as a Source Data file.

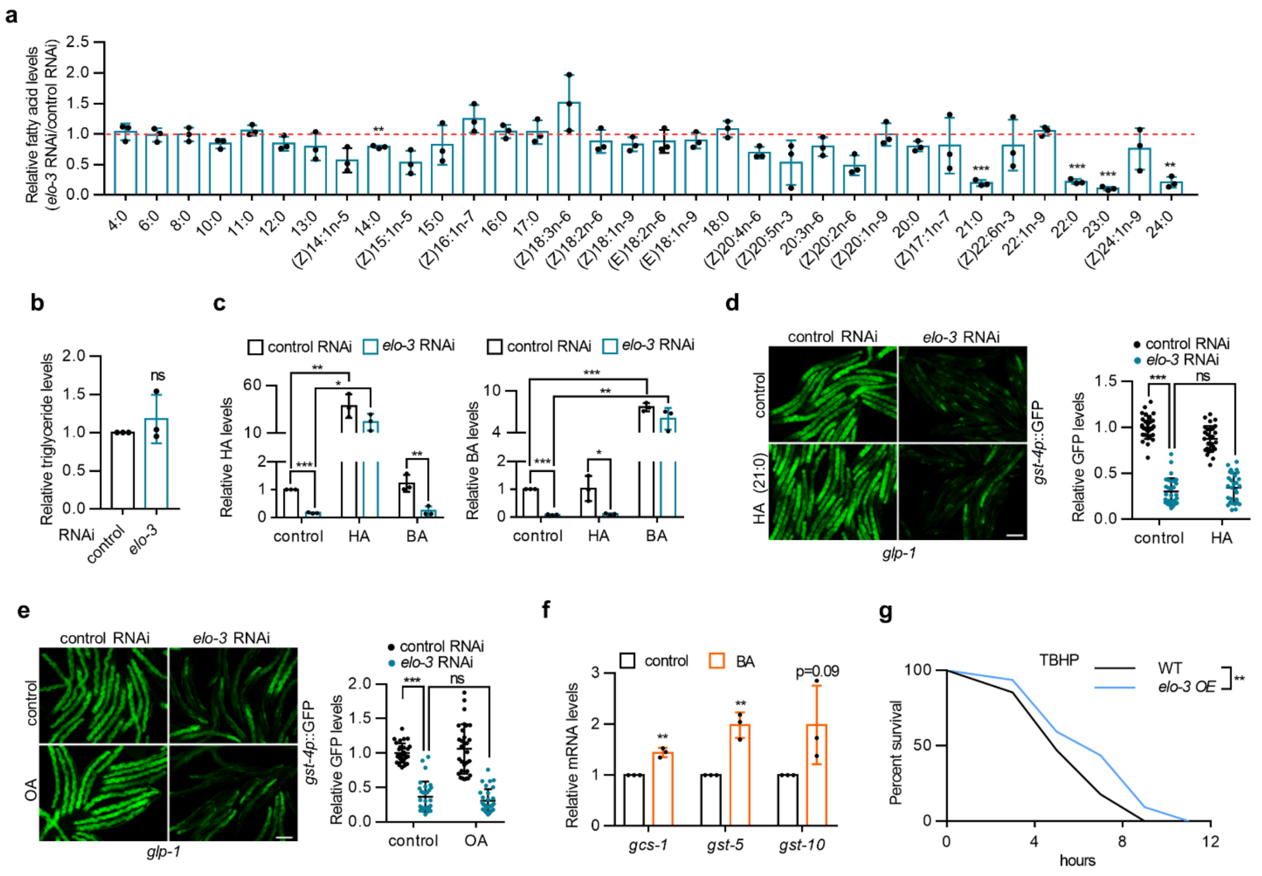

Supplementary Fig. 2. OA and HA are not involved in ELO-3-mediated SKN-1 activation. **(a)** GC-MS/MS analysis of fatty acid contents in wild-type animals treated with *elo-3* RNAi. n = 3 independent experiments. **(b)** Effects of *elo-3* RNAi on triglyceride contents. n = 3 independent experiments. **(c)** Effects of HA and BA supplementation on the contents of corresponding fatty acids. n = 3 independent experiments. **(d-e)** Effects of the supplementation of HA (21:0) **(d)** and OA **(e)** on the expression of *gst-4p*::GFP in *glp-1* mutants treated with *elo-3* RNAi. n = 30 animals. **(f)** Effects of BA supplementation on the expression of SKN-1 target genes. n = 3 independent experiments. **(g)** Effects of *elo-3* overexpressing on the TBHP resistance of wild-type worms. Data are represented as mean ± SD. **p* < 0.05, ***p* < 0.01, ****p* < 0.001. Fig. s2a was analyzed by multiple t test with correction for multiple comparisons using the Holm-Sidak method (****p* < 0.001, ***p* = 0.0020/0.0033 for 14:0/24:0). Figs. s2b and s2c were analyzed by unpaired two-tailed t test (For HA levels in panel c, ****p* < 0.001, ***p* = 0.0063/0.0076 for control vs HA/control RNAi BA vs *elo-3* RNAi BA, **p* = 0.0130; for BA levels in panel c, ****p* < 0.001, ***p* = 0.0027, **p* = 0.0258). Fig. s2d and e were analyzed by two-way ANOVA with Turkey's multiple comparison test (****p* < 0.001). Fig. s2f was analyzed by multiple t test with correction for multiple comparisons using the Holm-Sidak method (***p* = 0.0033/0.0050 for *gcs-1/gst-5*). See Supplementary Table 1 for statistical analysis and additional repeats of the survival assays **(g)** that was analyzed by log-rank (Mantel-Cox) test. Scale bar = 100 μm for panels d and e. Source data are provided as a Source Data file.

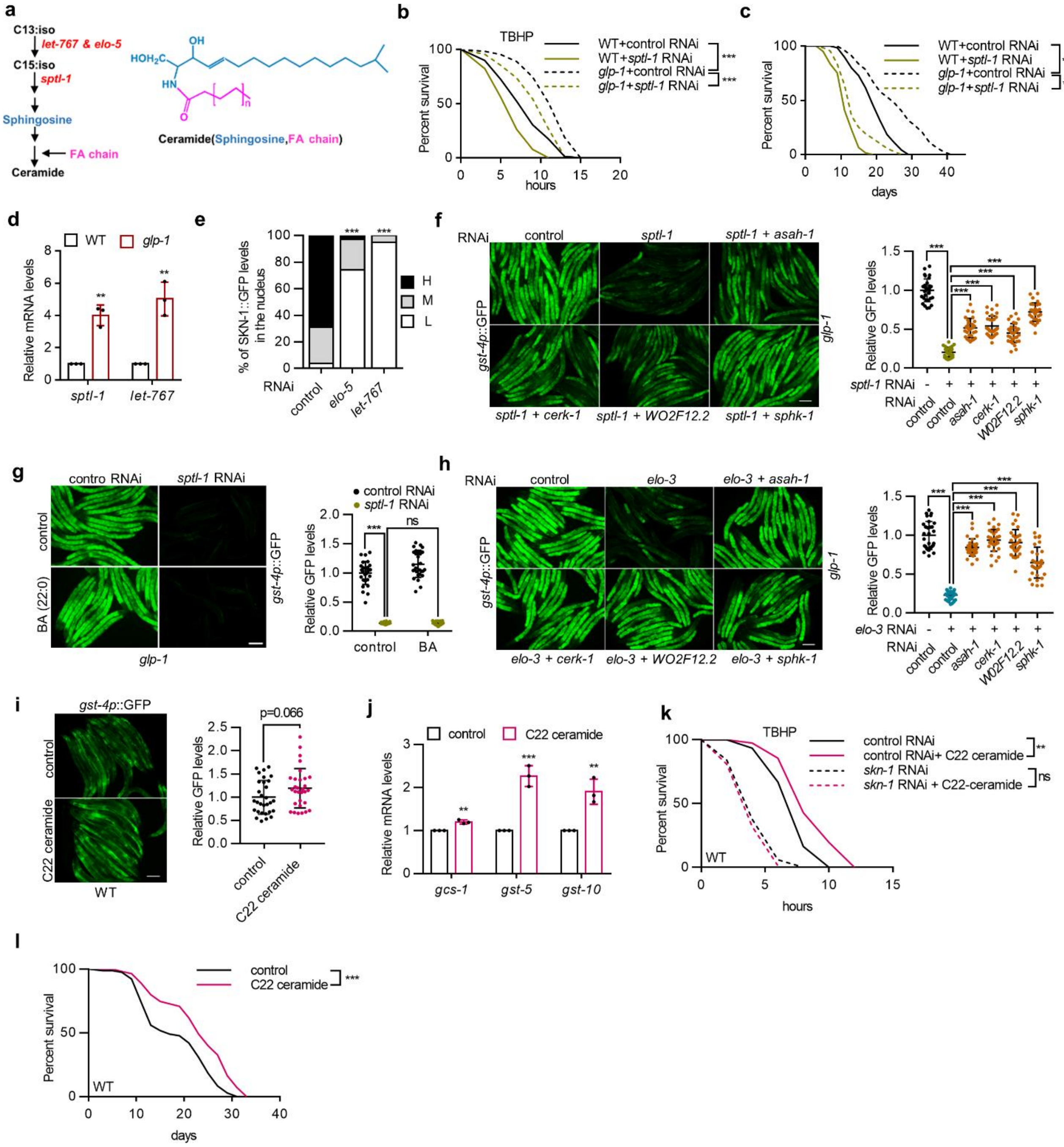

Supplementary Fig. 3. C22 ceramide regulates SKN-1 activation in germline-deficient worms. **(a)** Schematic of the biosynthetic pathway (left) and structure (right) of ceramide. **(b-c)** Effects of *sptl-1* RNAi on TBHP resistance **(b)** and longevity **(c)**. **(d)** The mRNA levels of *sptl-1* and *let-767* in *glp-1* mutants. n = 3 independent experiments. **(e)** Effects of *elo-5* and *let-767* RNAi on the SKN-1::GFP nuclear accumulation in *glp-1* mutants. n = 75/88/90 animals for control RNAi/*elo-5* RNAi/*let-767* RNAi. **(f)** The expression of *gst-4p::*GFP in *glp-1* mutants upon *sptl-1* RNAi treatment is rescued by the knockdowns of ceramide metabolic enzymes. n = 30 animals. **(g)** Effects of BA supplementation on the expression of *gst-4p*::GFP in *glp-1* mutants treated with *sptl-1* RNAi. n = 31

animals. **(h)** The expression of *gst-4p::*GFP in *glp-1* mutants upon *elo-3* knockdown is rescued by the knockdowns of ceramide metabolic enzymes. n = 30 animals. **(i-l)** Effects of C22 ceramide supplementation on the expression of *gst-4p*::GFP **(i)** (n = 30 animals) and SKN-1 targets **(j)** (n = 3 independent experiments)**,** TBHP resistance **(k)** and longevity **(l)** in wild-type worms. Data are represented as mean ± SD. *$p < 0.05$, **$p < 0.01$, ***$p < 0.001$. Figs. s3d and s3j were analyzed by multiple t test with correction for multiple comparisons using the Holm-Sidak method (***$p < 0.001$, **$p = 0.0024/0.0026$ for *sptl-1*/*let-767* in panel d, **$p = 0.0035/0.0058$ for *gcs-1*/*gst-10* in panel j). Fig. s3e was analyzed by Chi-square and Fisher's exact test (***$p < 0.001$). Figs. s3f and s3h were analyzed by one-way ANOVA with Turkey's multiple comparison test (***$p < 0.001$). Fig. s3g was analyzed by two-way ANOVA with Turkey's multiple comparison test (***$p < 0.001$). Fig s3i was analyzed by unpaired two-tailed t test. See Supplementary Table 1 and 2 for statistical analysis and additional repeats of the survival assays **(k, i)** that were analyzed by log-rank (Mantel-Cox) test. Scale bar = 100 μm for panels f, g, h and i. Source data are provided as a Source Data file.

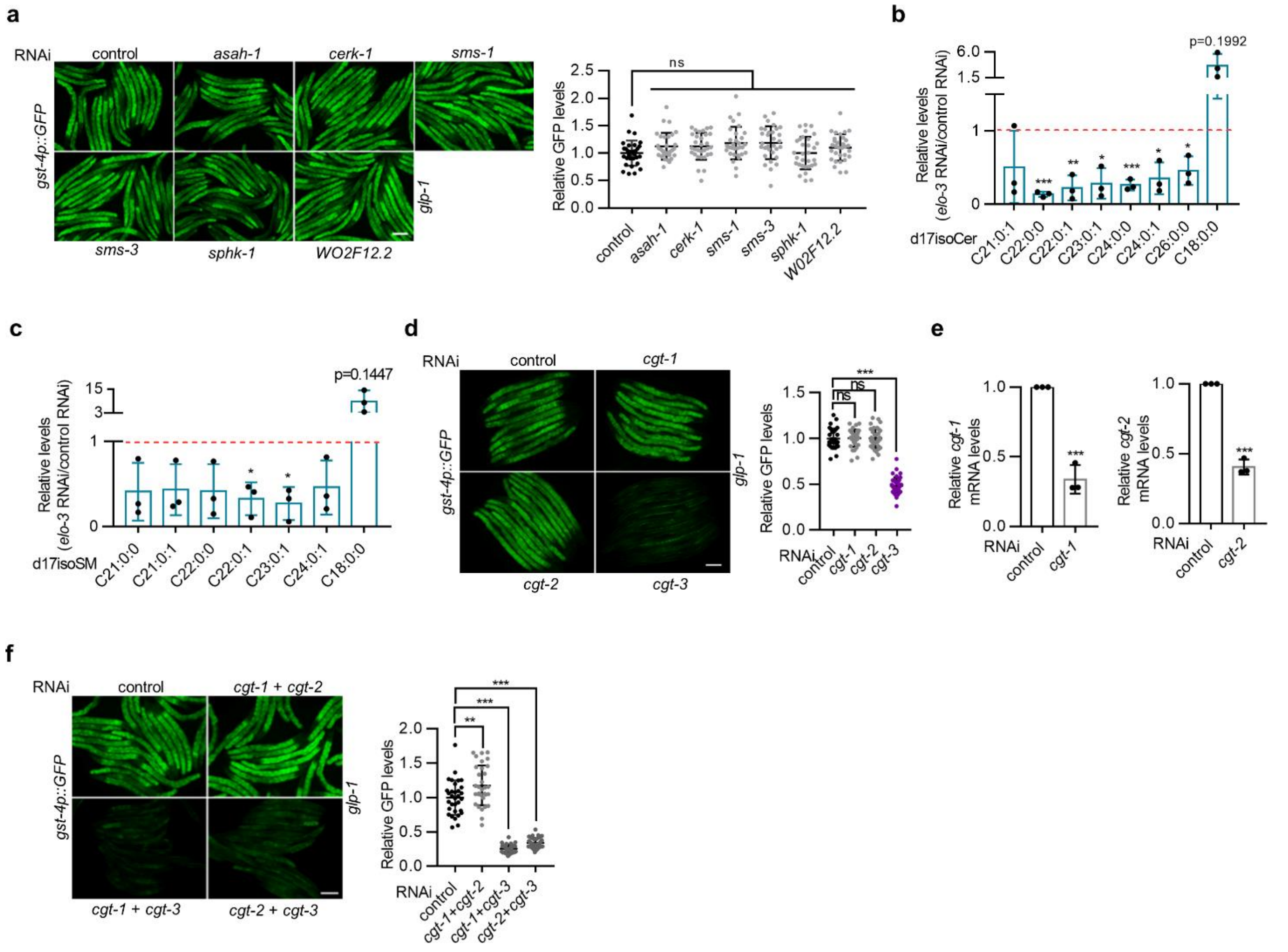

Supplementary Fig. 4. CGT-3 is critical for SKN-1 activation in germline-deficient animals. (a) Effects of RNAi targeting ceramide metabolic genes on the expression of *gst-4p*::GFP in *glp-1* mutants. n = 31 animals. **(b-c)** Effects of *elo-3* RNAi on the contents of major ceramides (d17isoCer) **(b)** and sphingomyelins (d17isoSM) **(c)**. n = 3 independent experiments. **(d)** Effects of *cgt-1* and *cgt-2* RNAi on the expression of *gst-4p*::GFP. n = 31/34/36/38 animals for control RNAi/*cgt-1* RNAi/*cgt-2* RNAi/*cgt-3* RNAi. **(e)** *cgt-1* and *cgt-2* RNAi efficiently knock down the mRNA levels of corresponding genes. n = 3 independent experiments. **(f)** Effects of combined RNAi targeting *cgt* genes on the expression of *gst-4p*::GFP in *glp-1* mutants. n = 32 animals. Data are represented as mean ± SD. *$p < 0.05$, **$p < 0.01$, ***$p < 0.001$. Figs. s4a, s4d and s4f were analyzed by one-way ANOVA with Turkey's multiple comparison test (***$p < 0.001$, **$p = 0.0028$ for panel f). Figs. s4b and s4c were analyzed by multiple t test with correction for multiple comparisons using the Holm-Sidak method (***$p < 0.001$, **$p = 0.0080$ in panel b, *$p = 0.0200/0.0263/0.0263$ for C23:0:1/C24:0:1/C26:0:0 in panel b, *$p = 0.0192/0.0288$ for C23:0:1/C22:0:1 in panel c). Fig. s4e was analyzed by unpaired two-tailed t test (***$p < 0.001$). Scale bar = 100 µm. Source data are provided as a Source Data file.

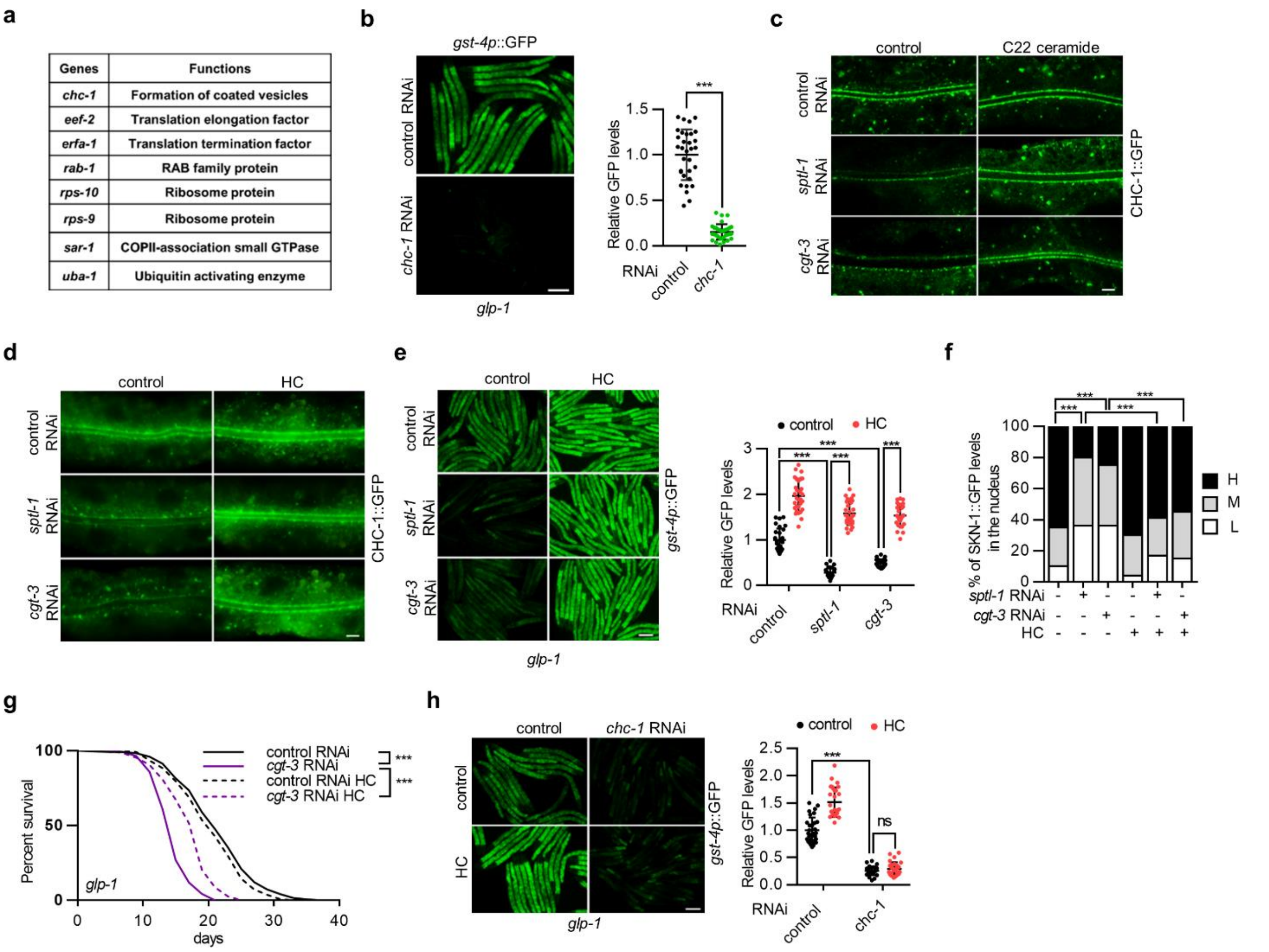

Supplementary Fig. 5. Clathrin mediates the effects of C22 GlcCer on SKN-1 activation and longevity. (a) Identified genes whose RNAi suppress *gst-4p*::GFP expression in *glp-1* mutants. **(b)** Effects of diluted *chc-1* RNAi on the expression of *gst-4p*::GFP in *glp-1* mutants. n = 32 animals. **(c)** Effects of C22 ceramide addition on the membrane localization of CHC-1::GFP in the intestine disrupted by *sptl-1* or *cgt-3* RNAi. **(d-g)** Effects of dietary high cholesterol (HC) on the membrane localization of CHC-1::GFP in the intestine **(d)**, *gst-4p*::GFP expression **(e)** (n = 30 animals), SKN-1 nuclear occupancy **(f)** (n = 76/86/96/79/90/79 animals for control RNAi/*sptl-1* RNAi/*cgt-3* RNAi/control RNAi HC/*sptl-1* RNAi HC/*cgt-3* RNAi HC) and longevity **(g)** in worms treated with *cgt-3* or *sptl-1* RNAi. **(h)** Effects of HC on the expression of *gst-4p*::GFP in *chc-1* RNAi-treated worms. n = 30 animals. Data are represented as mean ± SD. ***$p < 0.001$. Fig. s5b was analyzed by unpaired two-tailed t test (***$p < 0.001$). Figs. s5e and s5h were analyzed by two-way ANOVA with Turkey's multiple comparison test (***$p < 0.001$). Fig. s5f was analyzed by Chi-square and Fisher's exact test (***$p < 0.001$). See Supplementary Table 2 for statistical analysis and additional repeats of the survival assays **(g)** that was analyzed by log-rank (Mantel-Cox) test. Scale bar = 100 µm for panels b, e and h, 12 µm for panels c and d. Source data are provided as a Source Data file.

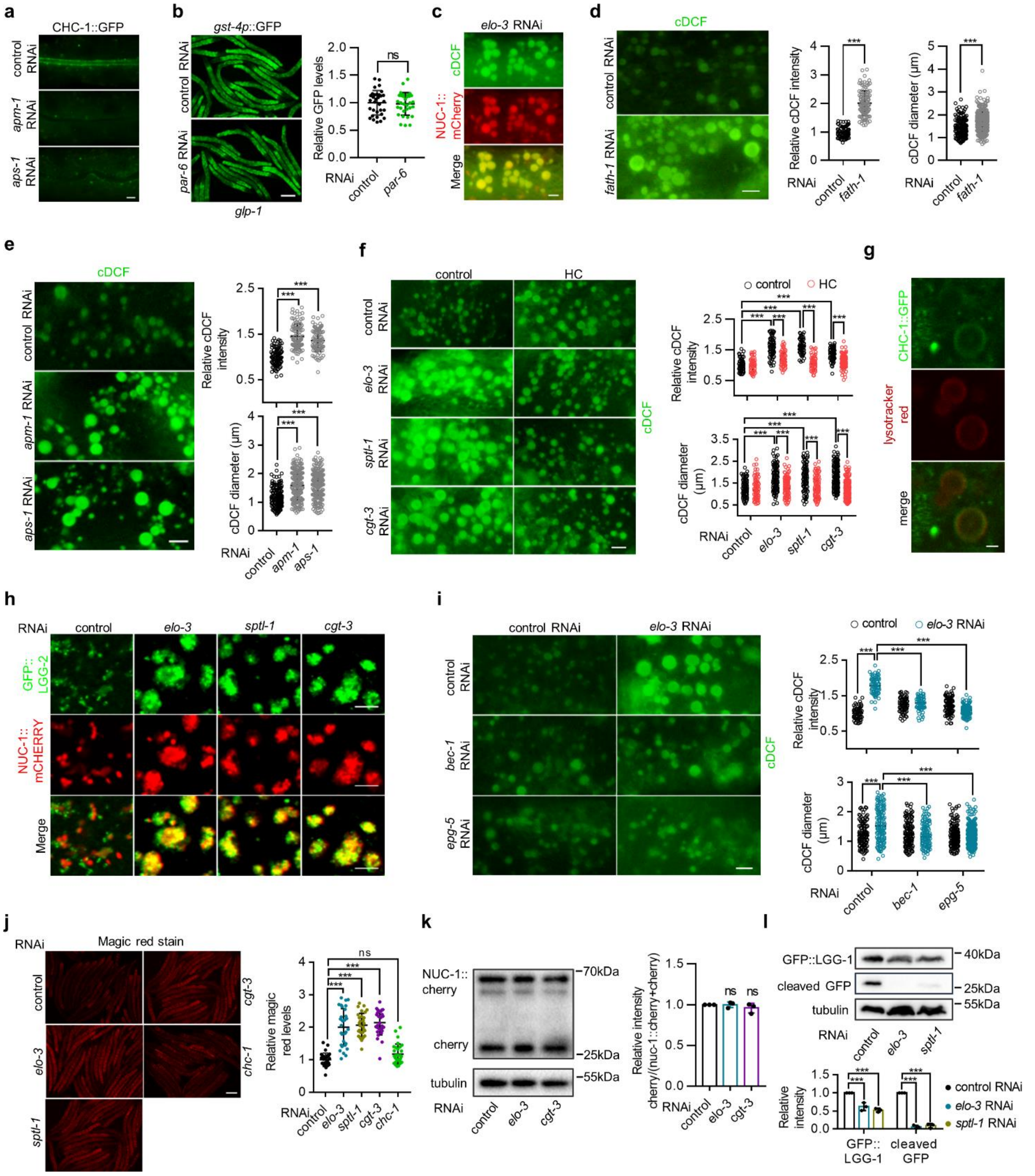

Supplementary Fig. 6. C22 GlcCer is required for lysosome homeostasis. (a) Effects of *aps-1* and *apm-1* RNAi on the membrane localization of CHC-1::GFP in the intestine. **(b)** Effects of *par-6* RNAi on the expression of *gst-4p*::GFP in *glp-1* mutants. n = 32 animals. **(c)** Colocalization of NUC-1::mCHERRY and cDCF signals in *elo-3* RNAi-treated animals. **(d)** Effects of *fath-1* on the acidity and size of lysosomes in the intestine of *glp-1* mutants. n = 108 (control RNAi) and 105 (*fath-1* RNAi) cDCF-positive granules for intensity analysis; n = 227 (control RNAi) and 256 (*fath-1* RNAi) cDCF-positive granules for size analysis. **(e)** Effects of *aps-1* and *apm-1* RNAi on the acidity and sizes of lysosomes in the intestine of *glp-1* mutants. n = 100 cDCF-positive granules for intensity

analysis; n = 205 (control RNAi), 211 (*apm-1* RNAi) and 202 (*aps-1* RNAi) cDCF-positive granules for size analysis. **(f)** Dietary HC reverses the lysosome phenotypes in animals treated with *elo-3*, *sptl-1* or *cgt-3* RNAi. n = 100 cDCF-positive granules for intensity analysis; n = 202(control RNAi), 208(*elo-3* RNAi, *sptl-1* RNAi, control RNAi HC, *sptl-1* RNAi HC and *cgt-3* RNAi HC), 200(*cgt-3* RNAi) and 203 (*elo-3* RNAi HC) cDCF-positive granules for size analysis. **(g)** Expression of CHC-1::GFP one the membrane of lysosomes stained by LysoTracker red in the intestine. **(h)** Colocalization of GFP::LGG-2 and NUC-1::mCHERRY in the hypodermis of animals treated with RNAi targeting the C22 GlcCer biosynthetic genes. **(i)** Effects of *bec-1* and *epg-5* RNAi on lysosome phenotypes in the intestine of *elo-3* knockdown worms. n = 101 cDCF-positive granules for intensity analysis; n = 218(control RNAi), 211(*elo-3* RNAi), 213(*epg-5* RNAi), 217(*elo-3* + *epg-5* RNAi), 204(*bec-1* RNAi) and 205(*elo-3* + *bec-1* RNAi) cDCF-positive granules for size analysis. **(j)** The activity of lysosomes measured by Magic Red Cathepsin assay in animals treated with *elo-3, sptl-1, cgt-3* or *chc-1* RNAi. n = 30 animals. **(k)** Effects of *elo-3* and *cgt-3* RNAi on lysosome activity as measured by NUC-1::mCHERRY cleavage. n = 3 independent experiments. **(l)** Effects of *elo-3* and *sptl-1* RNAi on autophagic degradation as measured by GFP::LGG-1 cleavage. n = 3 independent experiments. Data are represented as mean ± SD. *$p < 0.05$, ***$p < 0.001$. Figs. s6b and s6d were analyzed by unpaired two-tailed t test (***$p < 0.001$). Figs. s6e, s6j and s6k were analyzed by one-way ANOVA with Turkey's multiple comparison test (***$p < 0.001$). Figs. s6f and s6i were analyzed by two-way ANOVA with Turkey's multiple comparison test (***$p < 0.001$). Fig. s6l was analyzed by two-way ANOVA with Sidak's multiple comparisons test (***$p < 0.001$). Scale bar = 12 μm for panel a, 100 μm for panels b and j, 3 μm for panels c, d, e, f, h and i, 1 μm for panel g. Source data are provided as a Source Data file.

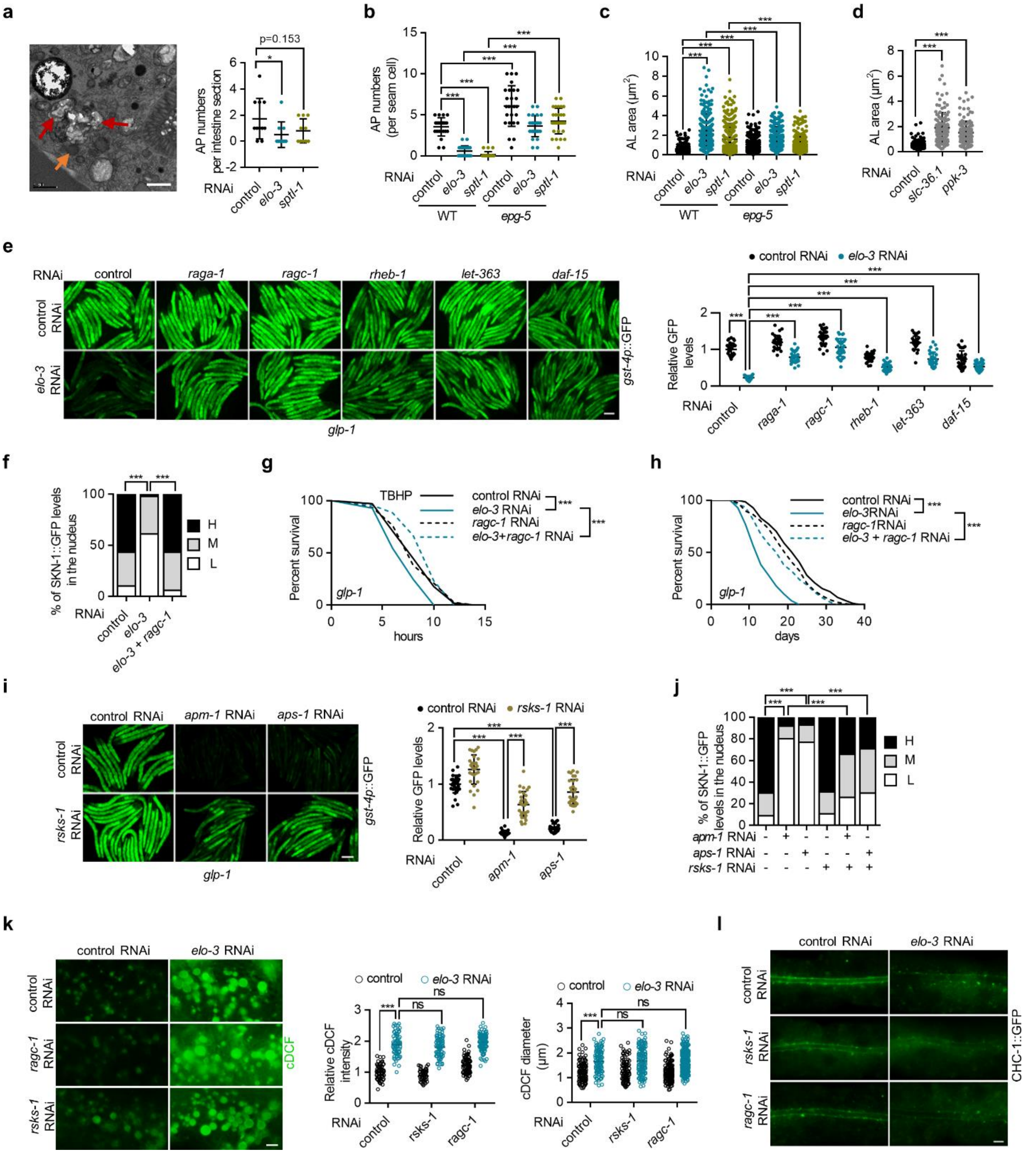

Supplementary Fig. 7. TOR suppression is required for C22 GlcCer-mediated SKN-1 activation and lifespan extension. (a) Effects of *elo*-*3* and *sptl-1* RNAi on AP numbers in the intestine of *glp-1* mutants as revealed by EM analysis. Red arrows indicate ALs and orange arrow indicates AP in the representative image (left panel). n = 11(control RNAi) and 10(*elo*-*3* and *sptl-1* RNAi) intestine sections. **(b-c)** Effects of *elo-3* and *sptl-1* RNAi on the number of APs in seam cells **(b)** (n = 24/27/20/26/26/29 seam cells for WT control RNAi/WT *elo-3* RNAi/WT *sptl-1* RNAi/*epg-5* control RNAi/*epg-5 elo-3* RNAi/*epg-5 sptl-1* RNAi) and size of ALs in the hypodermis **(c)** (n = 230/223/248/260/240/260 ALs for WT control RNAi/WT *elo-3* RNAi/WT *sptl-1* RNAi/*epg-5* control RNAi/ *epg-5 elo-3* RNAi/*epg-5 sptl-1* RNAi) in *epg-5* mutants as measured by yellow and

red signals from mCHERRY::GFP::LGG-1, respectively. **(d)** Effects of *slc-36.1* and *ppk-3* RNAi on the size of ALs as measured by the red fluorescence of mCHERRY::GFP::LGG-1. n = 125 (control RNAi), 129 (*slc-36.1* RNAi) and 130 (*ppk-3* RNAi) ALs. **(e)** Effects of RNAi targeting TOR components on the expression of *gst-4p*::GFP in *glp-1* mutants with *elo-3* knockdown. n = 30 animals. **(f-h)** Effects of *ragc-1* RNAi on the SKN-1 nuclear occupancy **(f)** (n = 83/74/81 animals for control RNAi/*elo-3* RNAi/*elo-3* + *ragc-1* RNAi), TBHP resistance **(g)** and lifespan **(h)** in *glp-1* mutants with *elo-3* knockdown. **(i-j)** Effects of *rsks-1* RNAi on the expression of *gst-4p*::GFP **(i)** (n = 30 animals) and SKN-1 nuclear occupancy **(j)** (n = 81/79/86/88/99/79 animals for control RNAi/*apm-1* RNAi/*aps-1* RNAi/*rsks-1* RNAi/*apm-1* + *rsks-1* RNAi/*aps-1* + *rsks-1* RNAi) in worms with *aps-1* or *apm-1* knockdown. **(k-l)** Effects of *rsks-1* and *ragc-1* RNAi on the lysosome phenotypes **(k)** [n = 100 cDCF-positive granules for intensity analysis; n = 203 (control RNAi/*elo-3* + *ragc-1* RNAi), 205 (*rsks-1* RNAi/*ragc-1* RNAi/*elo-3* + *rsks-1* RNAi) and 200 (*elo-3* RNAi) cDCF-positive granules for size analysis] and membrane localization of CHC-1::GFP **(l)** in the intestine of worms with *elo-3* knockdown. Data are represented as mean ± SD. **p* < 0.05, ***p* < 0.01, ****p* < 0.001. Figs. s7a and s7d were analyzed by one-way ANOVA with Turkey's multiple comparison test (****p* < 0.001, **p* = 0.0486 in panel a). Figs. s7b, s7c, s7e, s7i and s7k were analyzed by two-way ANOVA with Turkey's multiple comparison test (****p* < 0.001). Figs. s7f and j were analyzed by Chi-square and Fisher's exact test (****p* < 0.001). See Supplementary Table 1 and 2 for statistical analysis and additional repeats of the survival assays **(g, h)** that were analyzed by log-rank (Mantel-Cox) test. Scale bar = 1 μm for panel a, 100 μm for panels e and i, 3 μm for panel k, 12 μm for panel l. Source data are provided as a Source Data file.

Supplementary Table 1. Survival data for TBHP oxidative stress. Results shown are representative of at least two independent experiments. Repeats 1 were graphed in Figures.

Figures	Strain/Treatment	Mean Lifespan ± SEM (hours)	Worms Censored/Total	P value
**1f**				
**Repeat 1**	WT control RNAi	7.89 ± 0.24	2/77	
	WT *elo-3* RNAi	6.87 ± 0.25	1/75	0.007[b]
	glp-1 control RNAi	9.89 ± 0.22	0/70	<0.001[a]
	glp-1 elo-3 RNAi	8.26 ± 0.27	1/73	<0.001[b]
**Repeat 2**	WT control RNAi	7 ± 0.26	1/56	
	WT *elo-3* RNAi	5.98 ± 0.19	1/73	0.001[b]
	glp-1 control RNAi	8.54 ± 0.21	0/70	<0.001[a]
	glp-1 elo-3 RNAi	6.37 ± 0.24	2/70	<0.001[b]
**Repeat 3**	WT control RNAi	8.05 ± 0.27	3/83	
	WT *elo-3* RNAi	7.56 ± 0.27	2/86	0.2338[b]
	glp-1 control RNAi	10.04 ± 0.3	1/86	<0.001[a]
	glp-1 elo-3 RNAi	8.04 ± 0.25	2/79	<0.001[b]
**2e**				
**Repeat 1**	*glp-1* control RNAi	6.34 ± 0.19	7/76	
	glp-1 elo-3 RNAi	4.38 ± 0.17	5/72	<0.001[b]
	glp-1 elo-3 RNAi + BA	6.24 ± 0.20	9/76	<0.001[c]
**Repeat 2**	*glp-1* control RNAi	7.49 ± 0.16	2/104	
	glp-1 elo-3 RNAi	6.07 ± 0.14	16/150	<0.001[b]
	glp-1 elo-3 RNAi + BA	7.21 ± 0.20	15/97	<0.001[c]
**Repeat 3**	*glp-1* control RNAi	5.18 ± 0.13	0/88	
	glp-1 elo-3 RNAi	4.37 ± 0.11	0/79	<0.001[b]
	glp-1 elo-3 RNAi + BA	5.91 ± 0.19	5/92	<0.001[c]
**2g**				
**Repeat 1**	WT control RNAi	5.26 ± 0.13	5/88	
	WT control RNAi + C22	6.44 ± 0.14	6/101	<0.001[c]
	WT *skn-1* RNAi	4.36 ± 0.11	5/90	<0.001[b]

	WT *skn-1* RNAi + C22	4.48 ± 0.11	5/95	0.4592[c]
**Repeat 2**	WT control RNAi	5.29 ± 0.12	12/100	
	WT control RNAi + C22	6.14 ± 0.12	1/99	<0.001[c]
	WT *skn-1* RNAi	4.58 ± 0.09	7/86	<0.001[b]
	WT *skn-1* RNAi + C22	4.28 ± 0.11	2/80	0.0291[c]
**Repeat 3**	WT control RNAi	6.43 ± 0.18	7/99	
	WT control RNAi + C22	7.31 ± 0.17	4/93	0.001[c]
	WT *skn-1* RNAi	3.56 ± 0.09	3/91	<0.001[b]
	WT *skn-1* RNAi + C22	3.64 ± 0.08	9/95	0.3888[c]
**3h**				
**Repeat 1**	*glp-1* control RNAi	8.13 ± 0.23	4/91	
	glp-1 elo-3 RNAi	6.64 ± 0.16	10/78	<0.001[b]
	glp-1 elo-3 RNAi + C22 ceramide	8.15 ± 0.21	4/94	<0.001[c]
**Repeat 2**	*glp-1* control RNAi	6.43 ± 0.22	6/96	
	glp-1 elo-3 RNAi	4.61 ± 0.16	10/107	<0.001[b]
	glp-1 elo-3 RNAi + C22 ceramide	7.66 ± 0.24	9/108	<0.001[c]
**Repeat 3**	*glp-1* control RNAi	6.85 ± 0.24	0/79	
	glp-1 elo-3 RNAi	5.91 ± 0.14	0/88	<0.001[b]
	glp-1 elo-3 RNAi + C22 ceramide	7.40 ± 0.24	0/80	<0.001[c]
**4f**				
**Repeat 1**	WT control RNAi	7.3 ± 0.24	2/71	
	WT *cgt-3* RNAi	6.39 ± 0.21	1/73	0.0035[b]
	glp-1 control RNAi	9.26 ± 0.27	2/69	<0.001[a]
	glp-1 cgt-3 RNAi	7.92 ± 0.2	1/74	<0.001[b]
**Repeat 2**	WT control RNAi	7.96 ± 0.21	0/71	
	WT *cgt-3* RNAi	6.86 ± 0.21	1/86	0.001[b]
	glp-1 control RNAi	8.86 ± 0.22	2/72	<0.001[a]
	glp-1 cgt-3 RNAi	7.62 ± 0.19	1/69	<0.001[b]

**Repeat 3**	WT control RNAi	8.39 ± 0.27	2/77	
	WT *cgt-3* RNAi	7 ± 0.25	1/80	0.001[b]
	glp-1 control RNAi	10.22 ± 0.24	2/79	<0.001[a]
	glp-1 cgt-3 RNAi	7.65 ± 0.31	2/72	<0.001[b]
**4h**				
**Repeat 1**	WT control RNAi	5.84 ± 0.23	8/81	
	WT control RNAi + C22 ceramide	6.77 ± 0.26	6/77	0.0055[c]
	WT *cgt-3* RNAi	4.74 ± 0.21	10/75	<0.001[b]
	WT *cgt-3* RNAi + C22 ceramide	4.77 ± 023	8/76	0.8749[c]
**Repeat 2**	WT control RNAi	7.54 ± 0.17	1/92	
	WT control RNAi + C22 ceramide	8.91 ± 0.23	2/79	<0.001[c]
	WT *cgt-3* RNAi	6.30 ± 0.18	0/79	<0.001[b]
	WT *cgt-3* RNAi + C22 ceramide	6.18 ± 0.17	0/90	0.5525[c]
**Repeat 3**	WT control RNAi	5.58 ± 0.16	7/125	
	WT control RNAi + C22 ceramide	6.26 ± 0.22	3/109	0.0070[c]
	WT *cgt-3* RNAi	4.34 ± 0.19	5/102	<0.001[b]
	WT *cgt-3* RNAi + C22 ceramide	4.77 ± 0.20	2/101	0.0984[c]
**7e**				
**Repeat 1**	*glp-1* control RNAi	10.07 ± 0.18	0/86	
	glp-1 elo-3 RNAi	8.38 ± 0.18	2/82	<0.001[b]
	glp-1 rsks-1 RNAi	10.08 ± 0.23	2/81	0.5725[b]
	glp-1 elo-3 + rsks-1 RNAi	10.37 ± 0.24	2/85	<0.001[d]
**Repeat 2**	*glp-1* control RNAi	7.46 ± 0.22	2/79	
	glp-1 elo-3 RNAi	6.29 ± 0.13	0/63	<0.001[b]
	glp-1 rsks-1 RNAi	6.83 ± 0.18	1/69	0.0225[b]
	glp-1 elo-3 + rsks-1 RNAi	7.18 ± 0.18	0/76	<0.001[d]

**Repeat 3**	*glp-1* control RNAi	10 ± 0.2	3/76	
	glp-1 elo-3 RNAi	7.91 ± 0.23	3/78	<0.001[b]
	glp-1 rsks-1 RNAi	10 ± 0.2	2/81	0.8014[b]
	glp-1 elo-3 + *rsks-1* RNAi	9.66 ± 0.23	1/83	<0.001[d]
**S2g**				
**Repeat 1**	control	6.01 ± 0.23	4/69	
	elo-3 OE	7.15 ± 0.28	1/65	0.001[a]
**Repeat 2**	control	6.48 ± 0.25	2/67	
	elo-3 OE	7.22 ± 0.31	2/65	0.021[a]
**Repeat 3**	control	6.56 ± 0.25	4/69	
	elo-3 OE	7.51 ± 0.32	2/65	0.0082[a]
**S3b**				
**Repeat 1**	WT control RNAi	8.28 ± 0.39	0/69	
	WT *sptl-1* RNAi	6.45 ± 0.29	3/68	0.0001[b]
	glp-1 control RNAi	11.61 ± 0.35	2/66	<0.001[a]
	glp-1 sptl-1 RNAi	9.74 ± 0.36	1/69	0.0001[b]
**Repeat 2**	WT control RNAi	6.48 ± 0.28	1/65	
	WT *sptl-1* RNAi	5.84 ± 0.23	2/73	0.052[b]
	glp-1 control RNAi	10.06 ± 0.28	0/70	<0.001[a]
	glp-1 sptl-1 RNAi	8.46 ± 0.23	1/79	<0.001[b]
**S3k**				
**Repeat 1**	WT control RNAi	6.43 ± 0.19	2/81	
	WT control RNAi + C22 ceramide	7.39 ± 0.24	1/70	0.0056[c]
	WT *skn-1* RNAi	5.49 ± 0.16	3/75	<0.001[b]
	WT *skn-1* RNAi + C22 ceramide	5.47 ± 0.16	5/81	0.9515[c]
**Repeat 2**	WT control RNAi	7.54 ± 0.17	1/92	
	WT control RNAi + C22 ceramide	8.91 ± 0.23	2/79	<0.001[c]
	WT *skn-1* RNAi	4.56 ± 0.18	0/82	<0.001[b]

	WT *skn-1* RNAi + C22 ceramide	4.26 ± 0.15	3/84	0.2278[c]
**S7g**				
**Repeat 1**	*glp-1* control RNAi	8.66 ± 0.22	2/109	
	glp-1 elo-3 RNAi	7.25 ± 0.19	2/81	<0.001[b]
	glp-1 ragc-1 RNAi	8.8 ± 0.27	2/80	0.6193[b]
	glp-1 elo-3 + *ragc-1* RNAi	9.48 ± 0.25	0/69	<0.001[d]
**Repeat 2**	*glp-1* control RNAi	7.37 ± 0.24	1/74	
	glp-1 elo-3 RNAi	5.77 ± 0.19	0/70	<0.001[b]
	glp-1 ragc-1 RNAi	6.55 ± 0.2	1/67	0.0081[b]
	glp-1 elo-3 + *ragc-1* RNAi	7.18 ± 0.26	2/76	<0.001[d]

[a] vs same RNAi
[b] vs same strain
[c] vs same strain and same RNAi
[d] vs *glp-1 elo-3* RNAi

Supplementary Table 2. Lifespan data. Results shown are representative of at least two independent experiments. Repeats 1 were graphed in Figures.

Figures	Strain/Treatment	Mean Lifespan ± SEM (hours)	Worms Censored/Total	P value
**1g**				
**Repeat 1**	*glp-1* control RNAi	25.21 ± 0.71	10/77	
	glp-1 elo-3 RNAi	15.87 ± 0.30	9/81	<0.001[b]
**Repeat 2**	*glp-1* control RNAi	20.08 ± 0.6	6/73	
	glp-1 elo-3 RNAi	13.56 ± 0.38	7/81	<0.001[b]
**Repeat 3**	*glp-1* control RNAi	22.39 ± 0.83	6/64	
	glp-1 elo-3 RNAi	12.64 ± 0.4	7/76	<0.001[b]
**1h**				
**Repeat 1**	WT control RNAi	16.58 ± 0.76	5/67	
	WT *elo-3* RNAi	15.68 ± 0.69	4/67	0.0985[b]
**Repeat 2**	WT control RNAi	15.52 ± 0.58	9/72	
	WT *elo-3* RNAi	15.06 ± 0.46	4/68	0.2403[b]
**Repeat 3**	WT control RNAi	15.46 ± 0.6	4/61	

	WT *elo-3* RNAi	15.63 ± 0.5	7/71	0.8461[b]
**4g**				
**Repeat 1**	WT control RNAi	18.28 ± 0.53	6/79	
	WT *cgt-3* RNAi	12.74 ± 0.45	8/62	<0.001[b]
	glp-1 control RNAi	25.39 ± 0.92	8/87	<0.001[a]
	glp-1 cgt-3 RNAi	13.89 ± 0.48	8/69	<0.001[b]
**Repeat 2**	WT control RNAi	19.45 ± 0.51	8/84	
	WT *cgt-3* RNAi	14.55 ± 0.35	10/92	<0.001[b]
	glp-1 control RNAi	25.31 ± 0.98	13/79	<0.001[a]
	glp-1 cgt-3 RNAi	14.05 ± 0.41	8/86	<0.001[b]
**Repeat 3**	WT control RNAi	19.64 ± 0.54	12/100	
	WT *cgt-3* RNAi	14.41 ± 0.33	6/96	<0.001[b]
	glp-1 control RNAi	24.92 ± 1.03	14/77	<0.001[a]
	glp-1 cgt-3 RNAi	13.77 ± 0.41	8/89	<0.001[b]
**5c**				
**Repeat 1**	WT control RNAi	18.15 ± 0.65	7/65	
	WT *chc-1* RNAi	7.92 ± 0.18	10/82	<0.001[b]
	glp-1 control RNAi	23.99 ± 1.12	6/72	<0.001[a]
	glp-1 chc-1 RNAi	6.75 ± 0.14	6/83	<0.001[b]
**Repeat 2**	WT control RNAi	18.53 ± 0.56	7/81	
	WT *chc-1* RNAi	7.47 ± 0.24	8/81	<0.001[b]
	glp-1 control RNAi	24 ± 0.99	8/76	<0.001[a]
	glp-1 chc-1 RNAi	6.35 ± 0.14	5/89	<0.001[b]
**Repeat 3**	WT control RNAi	18.37 ± 0.55	3/85	
	WT *chc-1* RNAi	7.54 ± 0.24	6/76	<0.001[b]
	glp-1 control RNAi	24.08 ± 1.13	4/58	<0.001[a]
	glp-1 chc-1 RNAi	6.62 ± 0.13	10/100	<0.001[b]
**5h**				
**Repeat 1**	*glp-1* control RNAi	22.39 ± 0.83	7/64	
	glp-1 elo-3 RNAi	11.78 ± 0.3	6/74	<0.001[b]

	glp-1 control RNAi HC	21.04 ± 0.61	4/79	0.0799[c]
	glp-1 elo-3 RNAi HC	16.12 ± 0.61	15/68	<0.001[c]
**Repeat 2**	*glp-1* control RNAi	20.89 ± 0.62	6/73	
	glp-1 elo-3 RNAi	12.64 ± 0.4	7/76	<0.001[b]
	glp-1 control RNAi HC	20.24 ± 0.62	5/69	0.4665[c]
	glp-1 elo-3 RNAi HC	15.1 ± 0.59	15/71	<0.001[c]
**Repeat 3**	*glp-1* control RNAi	20.98 ± 0.64	7/73	
	glp-1 elo-3 RNAi	13.46 ± 0.4	8/74	<0.001[b]
	glp-1 control RNAi HC	19.35 ± 0.57	7/70	0.0339[c]
	glp-1 elo-3 RNAi HC	15.5 ± 0.68	18/62	<0.001[c]
**7f**				
**Repeat 1**	*glp-1* control RNAi	23.25 ± 1	3/73	
	glp-1 elo-3 RNAi	14.48 ± 0.65	8/84	<0.001[b]
	glp-1 rsks-1 RNAi	22.12 ± 0.92	9/82	0.2666[b]
	glp-1 elo-3 + rsks-1 RNAi	23.09 ± 0.8	6/82	<0.001[d]
**Repeat 2**	*glp-1* control RNAi	24.84 ± 0.89	7/74	
	glp-1 elo-3 RNAi	17.65 ± 0.53	7/81	<0.001[b]
	glp-1 rsks-1 RNAi	26.05 ± 0.75	10/95	0.5506[b]
	glp-1 elo-3 + rsks-1 RNAi	22.72 ± 0.72	7/78	<0.001[d]
**Repeat 3**	*glp-1* control RNAi	21.35 ± 0.71	4/80	
	glp-1 elo-3 RNAi	15.2 ± 0.52	5/87	<0.001[b]
	glp-1 rsks-1 RNAi	22.6 ± 0.81	9/84	0.1377[b]
	glp-1 elo-3 + rsks-1 RNAi	21.54 ± 0.77	6/76	<0.001[d]
**S1e**				
**Repeat 1**	*daf-2* control RNAi	25.03 ± 1.16	6/63	
	daf-2 elo-3 RNAi	20.25 ± 1.18	5/58	<0.001[b]
**Repeat 2**	*daf-2* control RNAi	28.56 ± 1.19	2/57	
	daf-2 elo-3 RNAi	22.08 ± 1.06	7/61	<0.001[b]
**S1f**				
**Repeat 1**	*eat-2* control RNAi	24.63 ± 0.9	2/66	

	eat-2 elo-3 RNAi	20.87 ± 0.7	8/66	<0.001[b]
**Repeat 2**	*eat-2* control RNAi	22.72 ± 0.84	6/ 63	
	eat-2 elo-3 RNAi	18.03 ± 0.69	5/66	<0.001[b]
**S3c**				
**Repeat 1**	WT control RNAi	19.29 ± 0.61	6/76	
	WT *sptl-1* RNAi	11.59 ± 0.39	8/69	<0.001[b]
	glp-1 control RNAi	25.3 ± 0.923	8/87	<0.001[a]
	glp-1 sptl-1 RNAi	14.26 ± 0.59	8/75	<0.001[b]
**Repeat 2**	WT control RNAi	16.88 ± 0.55	6/69	
	WT *sptl-1* RNAi	10.57 ± 0.43	4/57	<0.001[b]
	glp-1 control RNAi	19.18 ± 0.76	5/67	0.0028[a]
	glp-1 sptl-1 RNAi	14.4 ± 0.58	5/68	<0.001[b]
**S3l**				
**Repeat 1**	WT control RNAi	18.38 ± 0.84	16/88	
	WT control RNAi + C22 ceramide	22.58 ± 0.99	5/61	<0.001[c]
**Repeat 2**	WT control RNAi	15.71 ± 0.57	6/129	
	WT control RNAi + C22 ceramide	20.39 ± 0.70	13/127	<0.001[c]
**S5g**				
**Repeat 1**	*glp-1* control RNAi	22.39 ± 0.83	7/64	
	glp-1 cgt-3 RNAi	14.75 ± 0.34	2/73	<0.001[b]
	glp-1 control RNAi HC	21.04 ± 0.61	4/79	0.0799[c]
	glp-1 cgt-3 RNAi HC	16.57 ± 0.54	6/64	<0.001[c]
**Repeat 2**	*glp-1* control RNAi	20.89 ± 0.62	6/73	
	glp-1 cgt-3 RNAi	14.69 ± 0.31	4/74	<0.001[b]
	glp-1 control RNAi HC	20.24 ± 0.62	5/69	0.4665[c]
	glp-1 cgt-3 RNAi HC	17.93 ± 0.46	6/62	<0.001[c]
**S7h**				
**Repeat 1**	*glp-1* control RNAi	22.27 ± 0.86	3/73	
	glp-1 elo-3 RNAi	13.51 ± 0.48	5/84	<0.001[b]

	glp-1 ragc-1 RNAi	20.31 ± 0.76	8/91	0.0766[b]
	glp-1 elo-3 + *ragc-1* RNAi	18.66 ± 0.79	6/80	<0.001[d]
**Repeat 2**	*glp-1* control RNAi	22.75 ± 0.91	6/78	
	glp-1 elo-3 RNAi	13.3 ± 0.47	7/85	<0.001[b]
	glp-1 ragc-1 RNAi	20.52 ± 0.76	7/92	0.0412[b]
	glp-1 elo-3 + *ragc-1* RNAi	19 ± 0.8	6/81	<0.001[d]

[a] vs same RNAi
[b] vs same strain
[c] vs same strain and same RNAi
[d] vs *glp-1 elo-3* RNAi

Supplementary Table 3. qPCR primers.

**Primers**	**Sequences**
elo-3 Forward	CATCTGTCGTGTATGTAG
elo-3 Reverse	TATGGTGTGACTTCCTAT
let-767 Forward	GTTCTTCTGTCGCCAATC
let-767 Reverse	TAGTTCTCTGAGTGCTATACG
sptl-1 Forward	TTCAGGACTCGGATATTG
sptl-1 Reverse	ACTCTACTTGGTTCTTCAT
gcs-1 Forward	ATCTCTACCATCGTCTGA
gcs-1 Reverse	TCTTCGGCTTGTAACTTAT
gst-5 Forward	TTCCTACAAGTTGACCTA
gst-5 Reverse	TGACTCTATTATCCTCGTAT
gst-10 Forward	CATCCGTCTTCTGTTCCT
gst-10 Reverse	AACTCTTGCCATTCATTCC
cdc-42 Forward	AGCCATACACATTAGGAT
cdc-42 Reverse	AGAAGCAAACAAGAAACA
