# Supplementary material for: Saturated very long chain fatty acid configures glycosphingolipid for lysosome homeostasis in long-lived C. elegans
Source: Nat Commun. 2021 Aug 20;12:5073. doi: 10.1038/s41467-021-25398-6 (PMC8379269; doi:10.1038/s41467-021-25398-6)
Supplement: Supplementary file 3 — Reporting Summary [file 41467_2021_25398_MOESM3_ESM.pdf]

nature research

Corresponding author(s): DBPR NCOMMS-20-15669C

Last updated by author(s): Jun 21, 2021

Reporting Summary

Nature Research wishes to improve the reproducibility of the work that we publish. This form provides structure for consistency and transparency in reporting. For further information on Nature Research policies, see our Editorial Policies and the Editorial Policy Checklist.

Statistics

For all statistical analyses, confirm that the following items are present in the figure legend, table legend, main text, or Methods section.

n/a	Confirmed	
☐	☒	The exact sample size (*n*) for each experimental group/condition, given as a discrete number and unit of measurement
☒	☐	A statement on whether measurements were taken from distinct samples or whether the same sample was measured repeatedly
☐	☒	The statistical test(s) used AND whether they are one- or two-sided<br>*Only common tests should be described solely by name; describe more complex techniques in the Methods section.*
☐	☒	A description of all covariates tested
☐	☒	A description of any assumptions or corrections, such as tests of normality and adjustment for multiple comparisons
☐	☒	A full description of the statistical parameters including central tendency (e.g. means) or other basic estimates (e.g. regression coefficient) AND variation (e.g. standard deviation) or associated estimates of uncertainty (e.g. confidence intervals)
☐	☒	For null hypothesis testing, the test statistic (e.g. *F*, *t*, *r*) with confidence intervals, effect sizes, degrees of freedom and *P* value noted<br>*Give P values as exact values whenever suitable.*
☒	☐	For Bayesian analysis, information on the choice of priors and Markov chain Monte Carlo settings
☒	☐	For hierarchical and complex designs, identification of the appropriate level for tests and full reporting of outcomes
☒	☐	Estimates of effect sizes (e.g. Cohen's *d*, Pearson's *r*), indicating how they were calculated

Our web collection on statistics for biologists contains articles on many of the points above.

Software and code

Policy information about availability of computer code

Data collection	Nikon NIS-Elements, Leica LAS X, CFX Maestro, GCMSsolution
Data analysis	Blots and fluorescent image intensity were analyzed by Image J 1.53e. CDCFDA image size was analyzed by Nikon NIS-Elements and autolysosome size was analyzed by Leica LAS X. Statistical testing was performed by GraphPad Prism 8.

For manuscripts utilizing custom algorithms or software that are central to the research but not yet described in published literature, software must be made available to editors and reviewers. We strongly encourage code deposition in a community repository (e.g. GitHub). See the Nature Research guidelines for submitting code & software for further information.

Data

Policy information about availability of data

All manuscripts must include a data availability statement. This statement should provide the following information, where applicable:

- Accession codes, unique identifiers, or web links for publicly available datasets
- A list of figures that have associated raw data
- A description of any restrictions on data availability

The data supporting the findings from this study are available within the article and its supplementary information. Source data are provided with this paper. Any remaining raw data will be available from the corresponding author upon reasonable request.

Field-specific reporting

Please select the one below that is the best fit for your research. If you are not sure, read the appropriate sections before making your selection.

☒ Life sciences ☐ Behavioural & social sciences ☐ Ecological, evolutionary & environmental sciences

For a reference copy of the document with all sections, see nature.com/documents/nr-reporting-summary-flat.pdf

Life sciences study design

All studies must disclose on these points even when the disclosure is negative.

Sample size	Sample size was chosen based on the general standard in the C. elegans field. For fluorescent analysis n > 30 animals or n > 100 organelles. For lifespan analysis n = 70-120 animals. For statistical analysis of qPCR, immunoblotting and metabolic data, 3 independent biological repeats were performed.
Data exclusions	No data were excluded.
Replication	Lifespan data were repeated three times for main figures and at least twice for supplementary figures. Other data were repeated at least three times, with similar results.
Randomization	Worms were placed into each group randomly.
Blinding	The investigators were not blinded during data collection. For lifespan analysis, the strong phenotype of RNAi-treated worms makes blinding surplus. The lifespan assay was also carefully repeated by different persons, with similar results. For imaging experiments, worms for imaging were collected randomly, and images were acquired and analyzed by commercial software. For qPCR, immunoblotting and metabolic analysis, data were collected and analyzed by commercial softwares, and multiple repeats were conducted with similar results.

Reporting for specific materials, systems and methods

We require information from authors about some types of materials, experimental systems and methods used in many studies. Here, indicate whether each material, system or method listed is relevant to your study. If you are not sure if a list item applies to your research, read the appropriate section before selecting a response.

Materials & experimental systems

n/a	Involved in the study	
☐	☒	Antibodies
☒	☐	Eukaryotic cell lines
☒	☐	Palaeontology and archaeology
☐	☒	Animals and other organisms
☒	☐	Human research participants
☒	☐	Clinical data
☒	☐	Dual use research of concern

Methods

n/a	Involved in the study	
☒	☐	ChIP-seq
☒	☐	Flow cytometry
☒	☐	MRI-based neuroimaging

Antibodies

Antibodies used	GFP (Santa Cruz, SC-9996), mCherry (Sungene Biotech, KM8017), p-P70 S6K (cell signaling, #9205), tubulin (sigma, T9026)
Validation	GFP antibody (Santa Cruz, SC-9996) was validated by PMID 33811077 and by manufacturer (https://www.scbt.com/zh/p/gfp-antibody-b-2): GFP Antibody (B-2) is a high quality monoclonal GFP antibody (also designated Green Fluorescent Protein antibody) suitable for the detection of the GFP protein. .<br>mCherry antibody (Sungene Biotech, KM8017) was validated by manufacturer's website (http://www.sungenebiotech.com/index.php?m=Product&a=product_xq&catid=2&proid=54&prid=293&pid=712&id=1537): Western blot analysis of 28a-mCherry fusion protein with anti mCherry-tag mouse mAb (13A4).<br>p-P70 S6K antidbody (cell signaling, #9205) was validated by PMID 29027899 and by manufacturer's website (https://www.cellsignal.com//products/primary-antibodies/phospho-p70-s6-kinase-thr389-antibody/9205): Phospho-p70 S6 Kinase (Thr389) Antibody detects endogenous levels of p70 S6 kinase only when phosphorylated at threonine 389.<br>tubulin antibody (sigma, T9026) was validated by PMID 22872865 and by manufacturer's website (https://www.sigmaaldrich.cn/CN/en/product/sigma/t9026?context=product): The antibody is specific for α-tubulin in immunoblotting assays.

Animals and other organisms

Policy information about studies involving animals; ARRIVE guidelines recommended for reporting animal research

Laboratory animals	C. elegans strains used in this study were listed in Methods. Day 1 adult hermaphrodites were used in this study.
Wild animals	Not involved
Field-collected samples	Not involved
Ethics oversight	no ethical approval is needed for C. elegan study.

Note that full information on the approval of the study protocol must also be provided in the manuscript.
